# Clinical spectrum, cardiac phenotypes, and outcomes of FHL1-related cardiomyopathies: a systematic review

**DOI:** 10.1186/s12872-026-06096-x

**Published:** 2026-06-17

**Authors:** Emanuele Bobbio, Martina Caiazza, Filomena Pisacane, Immacolata Viscovo, Alessandro Gentile, Emanuele Monda, Chiara De Falco, Daniela Esposito, Felice Borrelli, Mariangela Losi, Eduardo Bossone, Suet Nee Chen, Giulia Frisso, Paolo Calabrò, Giovanni Esposito, Raffaella Lombardi, Giuseppe Limongelli

**Affiliations:** 1 Inherited and Rare Cardiovascular Diseases, Department of Translational Medical Sciences, University of Campania ’Luigi Vanvitelli’, Monaldi Hospital, via Leonardo Bianchi 1, Naples, 80131 Italy; 2 UOC Patologia Clinica, AORN dei Colli, via Leonardo Bianchi 1, Naples, 80131 Italy; 3 Department of Advanced Biomedical Sciences, University of Naples Federico II, Via Pansini 5, Naples, 80131 Italy; 4 Division of Cardiology, University of Colorado School of Medicine, 12505 E. 16th Avenue, Anschutz Inpatient Pavilion 2, Aurora, CO 80045 USA; 5 Department of Internal Medicine and Clinical Nutrition, Institute of Medicine, Sahlgrenska Academy, Gothenburg, Sweden; 6 Department of Endocrinology at Sahlgrenska University Hospital, University of Gothenburg, Blå Stråket 8, Gothenburg, 413 45 Sweden; 7 Department of Public Health, University of Naples Federico II, Via Pansini 5, Naples, 80131 Italy; 8 Heart Centre of Excellence, King Faisal Specialist Hospital & Research Center, Riyadh, Saudi Arabia; 9 Department of Molecular Medicine and Medical Biotechnology, University of Naples Federico II, Via Pansini 5, Naples, 80131 Italy; 10 Department of Translational Medical Sciences, University of Campania Luigi Vanvitelli, Caserta, 81100 Italy; 11 Institute of Cardiovascular Science, University College London, Gower Street, London, WC1E 6BT UK; 12 Department of Cardiology, Sahlgrenska University Hospital, Gothenburg, Sweden; 13 Institute of Medicine, Sahlgrenska Academy, University of Gothenburg, Gothenburg, Sweden

**Keywords:** FHL1, Hypertrophic cardiomyopathy, Arrhythmia, Sudden cardiac death, Cardiogenetics, Heart failure

## Abstract

**Background:**

Mutations in the *Four-and-a-Half LIM Domains 1* (*FHL1*) gene are increasingly recognized as a rare cause of inherited cardiomyopathies, often associated with skeletal myopathy and adverse cardiac outcomes. The phenotypic spectrum and clinical implications of *FHL1* variants remain poorly defined.

**Objective:**

To systematically review published cases of *FHL1*-related cardiomyopathy and characterize the clinical, genetic, and pathological features.

**Methods:**

We conducted a systematic literature search in PubMed and EMBASE up to July 2025 using predefined criteria to identify studies reporting clinical cases of patients with *FHL1* mutations and cardiac involvement. Data on genotype, phenotype, cardiac and neuromuscular features, and clinical outcomes were extracted and synthesized.

**Results:**

Twenty-two studies were included, comprising 114 patients with pathogenic or likely pathogenic *FHL1* mutations. Most patients were male (69%), with a median age of onset of 18 (IQR 10–26) years. Cardiac involvement consisted in left ventricular hypertrophy (56%), followed by arrhythmias (51%), and conduction abnormalities (8%). The incidence of sudden cardiac death was 7%, and heart transplantation was reported in 5% of patients. Skeletal muscle involvement was present in 75%, ranging from mild contractures to more severe myopathic phenotypes with functional impairment. Creatine kinase levels were variably elevated. Truncating variants were reported in several severe cardiac presentations in young males, while isolated cardiac disease occurred with selected variants.

**Conclusions:**

*FHL1*-related cardiomyopathy is a rare but important diagnosis. Genetic testing should be considered in patients with cardiac hypertrophy and neuromuscular features. Further research is needed to define prognostic markers and guide management.

## Introduction

*Four-and-a-half LIM domains 1* (*FHL1*) gene is located on chromosome Xq26.3, and encodes for a Zink-finger protein which is abundantly expressed in striated muscle [1, 2]. Through alternative splicing, *FHL1* gives rise to three main isoforms (FHL1A, FHL1B, and FHL1C) which mediate critical protein–protein interactions involved in sarcomere assembly, mechanotransduction, and gene regulation [3]. FHL1A, the predominant isoform in adult skeletal and cardiac muscle, interacts with titin in the sarcomere and with ERK2 in the nucleus, contributing to muscle integrity and adaptation to mechanical stress [4, 5].

Originally identified in patients with reducing body myopathy (RBM) [6, 7], *FHL1* mutations have since been implicated in a broad phenotypic spectrum that includes X-linked myopathy with postural muscle atrophy (XMPMA), Emery–Dreifuss muscular dystrophy (EDMD)-like syndromes, and rigid spine myopathies. Several *FHL1* pathogenic variants have been associated with predominant or isolated cardiac involvement, most often hypertrophic cardiomyopathy (HCM), conduction system disease, ventricular arrhythmias, and sudden cardiac death (SCD) [2, 8–12]. In some male patients, cardiac symptoms may precede or even occur without skeletal muscle involvement, while female carriers often exhibit incomplete or milder phenotypes, consistent with X-linked inheritance [13, 14].

Variants frequently affect conserved cysteine residues which are essential for the structural support of the LIM domains, leading to toxic protein aggregation or loss of function. Certain variants, such as p.(Cys276Tyr) has been associated with HCM without skeletal muscle involvement [2], while others, such as the p.(Cys224Trp) variant, have been reported in subjects with XMPMA with or without association with cardiac conduction abnormalities or cardiomyopathy [15]. These findings suggest possible genotype–phenotype correlation.

Given the rarity of *FHL1*-related conditions and the wide variability in cardiac presentation, clinical recognition remains limited; furthermore, *FHL1* remains underrepresented in the cardiomyopathy genetic screening panels, and no consensus exists regarding screening, arrhythmia surveillance, or preventive implantable cardioverter defibrillator (ICD) implantation. A better understanding of the spectrum of cardiac involvement is essential for early diagnosis, risk stratification, and management [16].

This systematic review aims to define the clinical and genetic characteristics and outcome of *FHL1*-related cardiomyopathies by synthesizing data from existing literature.

## Methods

The protocol for this systematic review was developed in accordance with the preferred reporting items for systematic review and meta-analysis protocol (PRISMA-P). This systematic review is reported in conformity with the PRISMA guidelines [17] and was registered in PROSPERO (CRD420251117666).

### Literature search strategy

We conducted a comprehensive systematic review of the literature using the *PubMed* database and EMBASE (Elsevier) to identify published studies reporting on cardiac manifestations associated with pathogenic mutations in the *FHL1* gene. The search was performed using the following predefined query: “FHL1“[Title/Abstract] AND (“cardiomyopathy” OR “heart failure” OR “arrhythmia” OR “sudden cardiac death”) AND (“mutation” OR “variant”) AND (humans[Filter]) AND (english[lang]). No date restriction was applied to maximize the capture of all relevant literature. The most recent search was conducted on July 31, 2025.

In addition to the primary database search, we manually screened the reference lists of all eligible full-text articles, as well as recent reviews on *FHL1*-related myopathies and cardiomyopathies, to identify additional publications not captured by the initial query. In addition, studies identified through expert consultation were screened and considered for inclusion using the same predefined eligibility criteria. Duplicate articles were removed prior to screening.

### Study selection

Two authors (EB and RL) independently reviewed all retrieved records in a two-step selection process. In the first step, titles and abstracts were screened to determine preliminary eligibility based on relevance to the research question. Articles were excluded at this stage if they clearly lacked original clinical data, did not involve *FHL1* mutations, or focused exclusively on skeletal muscle involvement without mentioning cardiac features. In the second step, full-text versions of all potentially eligible articles were obtained and examined in detail to assess whether they met the predefined inclusion criteria.

Studies were eligible for inclusion if they met the following criteria: (1) original articles, case series, or case reports; (2) description of at least one *FHL1* pathogenic or likely pathogenic genetic variant, confirmed by genetic sequencing; (3) availability of clinical data describing cardiac manifestations, including but not limited to cardiomyopathy, arrhythmias, conduction abnormalities, heart failure, or sudden cardiac death; and (4) published in English.

Articles were excluded if they: (1) lacked primary patient-level data (e.g., reviews, commentaries); (2) described animal or in vitro models only; (3) reported variants of unknown significance without supportive evidence of pathogenicity; (4) reported only incidental, minimal, or non-characterized cardiac findings without sufficient clinical detail, as they did not allow standardized data extraction; or (5) described only skeletal muscle findings without any reference to cardiac phenotype. Any discrepancies in inclusion decisions between the two reviewers were resolved through discussion and consensus. When multiple publications from the same research groups or potentially overlapping cohorts were identified, studies were carefully evaluated to avoid duplication of patient data. In such cases, preference was given to the most comprehensive report or to studies providing unique and extractable cardiac data.

### Data extraction and synthesis

Detailed data were extracted from each of the included studies using a standardized extraction form. The form captured publication details (first author, year), study design, and detailed patient-level or family-level information, including: number of affected individuals, sex, age at symptom onset (neuromuscular and cardiac), genetic mutation(s) identified (including variant type and position), and whether the variant affected specific *FHL1* isoforms.

Clinical phenotypes were classified according to the terminology used in the source publications, including EDMD, RBM, XMPMA, and isolated HCM. Cardiac features such as left ventricular hypertrophy (LVH), systolic and diastolic dysfunction, conduction disease, arrhythmias (supraventricular or ventricular), pacemaker or ICD implantation, heart failure, heart transplant and SCD were recorded in detail.

Laboratory findings including serum creatine kinase (CK) levels, as well as muscle magnetic resonance imaging (MRI), electromyography, and histopathological findings from muscle biopsy or autopsy, were also extracted when available. Particular attention was paid to the description of reducing bodies, fiber atrophy, rimmed vacuoles, fibrosis, and abnormal protein aggregates at the histological analysis.

Data extraction was performed independently by two authors and cross-verified. Where only family-level summaries were available, data were aggregated as reported. No formal risk of bias assessment was performed given the descriptive nature of included studies. Due to heterogeneity in study designs and patient characteristics, quantitative synthesis or meta-analysis was not performed. Results are presented narratively and in structured tables, with a focus on recurrent patterns and genotype–phenotype correlations.

### Ethics

As this review is based on already published data and lacks contact with individual patients, there is no requirement for ethical approval or consent.

## Results

### Overview of included studies

**Fig. 1:**
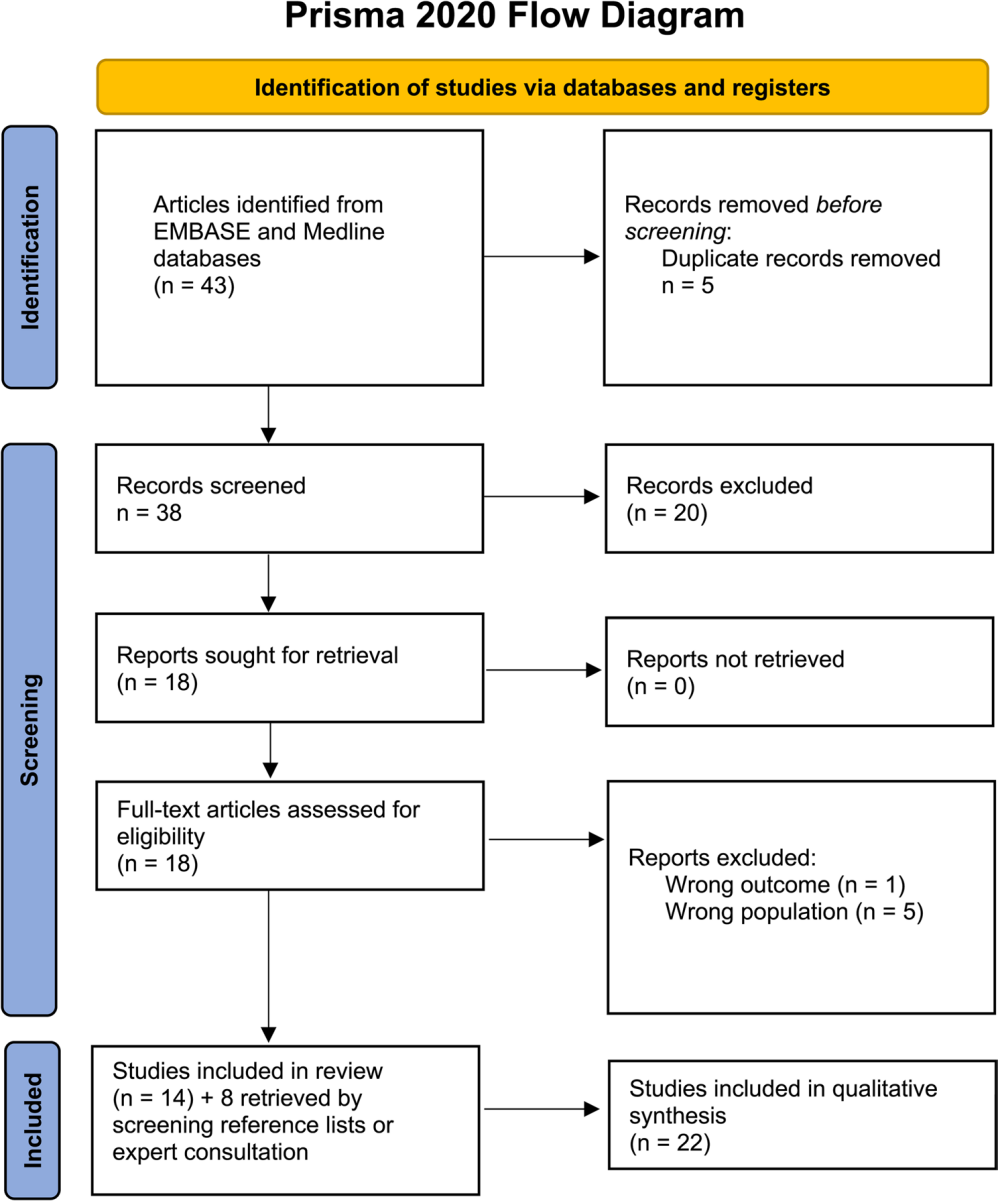
PRISMA flow diagram

A total of 38 studies were identified through the initial database search using the specified query, and 2 additional studies were retrieved by screening the reference lists of relevant articles. Furthermore, 6 additional studies were identified through expert consultation and supplementary manual screening, in line with the predefined eligibility criteria. After title and abstract screening, 20 records were excluded for not meeting inclusion criteria. Of the remaining 26 full-text articles, 22 met the inclusion criteria and were retained for final analysis (Fig. 1). One additional article represented an update of a previously included study and was treated as an extension of the original cohort rather than a separate case series [18, 19].

Across the 22 included studies, clinical and genetic data were available for 114 individuals carrying clinically relevant FHL1 mutations, including 79 males (69%) (Table 1). Among the male patients, all were symptomatic, while most females were either asymptomatic carriers or exhibited milder phenotypes. The median age of symptom onset, when reported, was approximately 18 years (Interquartile range, IQR: 10–26). Disease duration ranged widely depending on the study and the specific FHL1 mutation.

**Table 1:** Summary of included studies and patient demographics

First Author, Year	Patients with Clinically Relevant Mutation	Males (%)	Onset (yrs, median [IQR])	Skeletal muscle Involvement (%)	Cardiac Features (%)	Outcomes (%)
Binder et al., [15]	40	17 (43)	NA	All males with XMPMA, 3 females with mild contractures/rigid spine	Dyspnea (65), ECG: T-wave inversion (55), Q-waves (32), VT (5), LVH (50), diastolic dysfunction (67), spongiform myocardium (15)	No deaths or HTx reported. Non-sustained VT (12); CAD/stroke (5)
Borch et al., [20]	3	3 (100)	30 [17–38]	Mild to moderate myopathy; proximal/distal weakness (100)	HCM (100), non-sustained VT (33)	Death to acute HF at 24 y (33); ICD implantation (33)
D’Arcy et al., [8]	1	1 (100)	14 [NA]	Distal wasting and weakness (100)	HCM (100)	Pacemaker implantation at 16y
Finch et al., [21]	1	1 (100)	Childhood onset	Contractures, mild proximal weakness, rigid spine, scoliosis, motor delay (100%)	HCM (100)	Alive; progressive non-obstructive HCM; no arrhythmias
Friedrich et al., [2]	9	7 (78)	22 [14–49]	Mild symptoms in 2 subjects (22)	Asymmetric LVH (100), abnormal ECGs (67)	HTx (11)
Gaertner-Rommel et al., [22]	1	1 (100)	19 [NA]	None	Severe LVH	SCD at 19y
Gallego-Delgado et al., [23]	6	4 (67)	11 [8–38]	Mild cramps or no symptoms (83), normal exam (17)	AF (50), LVOTO (33)	SCD (17), aborted SCD (17), HTx (17), death due to pulmonary fibrosis (17)
Giucă et al., [24]	1	1 (100)	Cardiac symptoms at 40 yrs; myopathy symptoms appeared after	Wasting of shoulder and calf muscles, rigid spine (100)	HCM (100), AF (100)	Persistent HF symptoms.
Gossios et al., [25]	2	2 (100)	30 [NA]	Contractures only, no overt myopathy	LVH (100)	Alive, asymptomatic
Gueneau et al., [13]	7	7 (100)	14.7 [NA]	Weakness/Wasting Upper or lower limb (100), or axial/facial (71%); joint contractures (100)	Cardiac involvement in all: arrhythmias (57), conduction defects (28), LVH (57), biatrial dilation (14)	Three deaths (43): 2 SCD (31y and 51y), 1 due to respiratory insufficiency (37y).
Hartmannová et al., [9]	4	3 (75)	31 [17–55]	Mild hypotrophy in 1 male (25)	Non-obstructive HCM (100), AF (50), biatrial enlargement (75), abnormal ECG (100)	HTx (50) with 1 of them post-transplant death. Another patient had mild HF symptoms (25).
Knoblauch et al., [11]	9	9 (100)	10 [10–20]	Contractures (100), no weakness/atrophy	HCM (100), AF (11)	Two deaths (22): 1 cardiac/renal failure, 1 cardiopulmonary decompensation
Komagamine et al., [26]	3	1 (33)	18 [18–23]	Myopathy with rigid spine and asymmetric involvement (100)	Any cardiac abnormality (100): conduction abnormalities (67%), regional wall motion abnormalities (33), ECG abnormalities (33)	Respiratory insufficiency requiring nocturnal non-invasive ventilation (67); wheelchair dependence (67); no cardiac death reported
López Blázquez et al., [27]	1	1 (100)	9 [NA]	None	HCM with LVOTO (100)	Resuscitated cardiac arrest at 9y, ICD implanted. Appropriate shock 1y later
Nagaraj et al., [28]	1	1 (100)	7 [NA]	Myopathy with rigid spine and joint contractures (100)	HCM (100)	Alive, asymptomatic
Pen et al., [29]	4	4 (100)	NA	Myopathy with contractures and kyphoscoliosis (100)	Congenital heart defects (50), HCM (25)	Sudden death (25); cardiac arrest (25); remaining alive (50)
Quadrelli et al., [18](updated Xue [19])	6	6 (100)	Childhood onset	Muscle hypertrophy and weakness (100)	Unspecified cardiac abnormalities (100)	Death related to HF (50)
Schreckenbach et al., [16]	4	2 (50)	17.5 [7–32]	Proximal and distal asymmetric weakness (100)	Unspecified cardiac abnormalities leading to advances HF (50)	Death related to HF (50); death post-MI (25); wheelchair-bound (25).
San Román et al., [24]	6	4 (67)	44 [32–75]	All males displayed easy fatigability, thigh and calf myalgia or motor clumsiness (100). Unclear females.	Non-obstructive HCM (100), LVNC (17), systolic dysfunction (50), AF (67), prolonged QTc > 460 ms (83)	Cardiac arrest (17); HTx (33); progressive HF (50); ICD implantation (17)
Tiffin et al., [30]	3	3 (100)	NA (5 years in index case)	Myopathy with contractures and rigid spine (100)	Any cardiac abnormality (100): HCM (67), pulmonary valve stenosis (67), prolonged QTc (33)	Sudden cardiac death (67), ICD implantation (33), alive (33)
Willis et al., [31]	1	1 (100)	9 [NA]	Myopathy with muscle hypertrophy, rigid spine and contractures (100)	HCM (100), conduction abnormalities (100)	Alive, asymptomatic
Zhang et al., [32]	1	1 (100)	19 [NA]	Hypertrophy of all limb muscles without significant neurological findings	LVH with enlarged left atrium and moderately increased pulmonary systolic pressure	Asymptomatic to mild progression of HF

*AF* atrial fibrillation, *CAD *coronary artery disease, *ECG *electrocardiogram, *HF *heart failure, *HCM *hypertrophic cardiomyopathy, *HTx *heart transplantation, *ICD *implantable cardioverter-defibrillator, *IQR *interquartile range, *LVH *left ventricular hypertrophy, *LVNC *left ventricular non-compaction, *LVOTO *left ventricular outflow tract obstruction, *MI *myocardial infarction, *SCD *sudden cardiac death, *VT *ventricular tachycardia, *XMPMA *X-linked myopathy with postural muscle atrophy

### Epidemiology and genetic findings

**Fig. 2:**
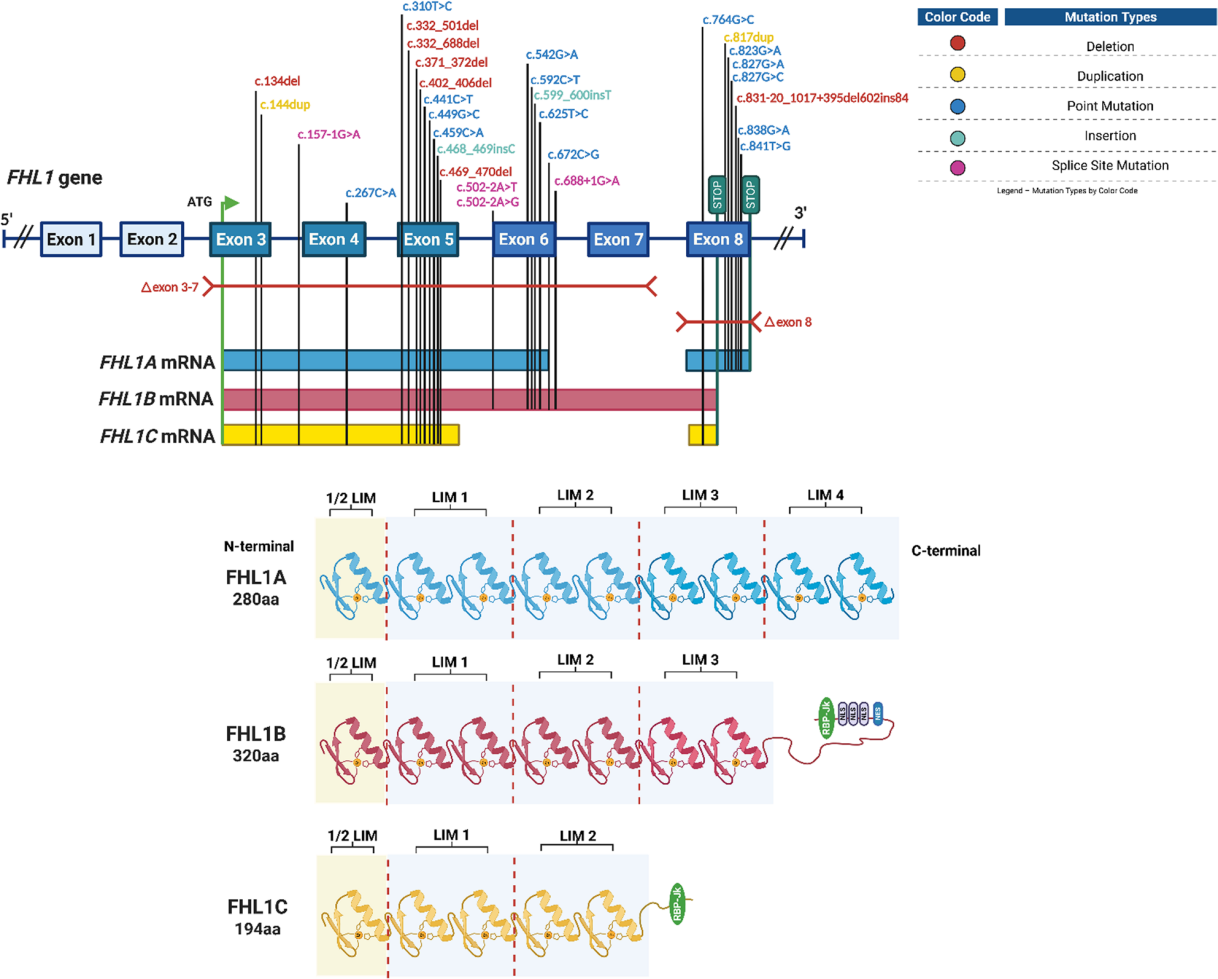
Schematic representation of the FHL1 gene, its main mRNA isoforms, and protein domain organization, with reported pathogenic variants. The upper panel shows the FHL1 (Four-and-a-half LIM domains 1) gene structure (exons 1–8) with the positions of pathogenic or likely pathogenic variants identified in the included studies, annotated using HGVS (Human Genome Variation Society) cDNA nomenclature. Variants are mapped to the exons they affect. The three major mRNA (messenger RNA) isoforms (FHL1A, FHL1B, and FHL1C) are shown below, with colored bars indicating their exon composition (blue: FHL1A, red: FHL1B, yellow: FHL1C).The lower panel depicts the corresponding protein structures, highlighting the LIM domains (half LIM, LIM1–LIM4) and their arrangement from N- to C-terminus. FHL1A contains four and a half LIM domains; FHL1B contains four and a half LIM domains plus a unique C-terminal extension with a NLS (nuclear localization signal); FHL1C contains two and a half LIM domains followed by a distinct C-terminal region. Zinc-binding sites are indicated by orange dots

Thirty-eight variants in the *FHL1* gene were reported across 22 studies [2, 8, 9, 11, 13, 15, 16, 18, 20–33] (Fig. 2). These included missense, nonsense, frameshift, splice-site variants, and large exonic deletions, predominantly affecting the LIM domains, which are essential for protein-protein interactions and sarcomeric anchoring (Table 2). The most prevalent mutation was c.672 C > G, p.(Cys224Trp), found in 29 of affected males (25% of all included patients) in a multicenter cohort and consistently linked to the XMPMA phenotype and prominent cardiac manifestations [15]. Other frequently reported missense mutations included c.625T > C, p.(Cys209Arg), c.764G > C, p.(Cys255Ser), and c.449G > C, p.(Cys150Ser), all targeting conserved cysteine residues critical for LIM domain structural stability [8, 13, 16].

**Table 2:** Summary of FHL1 variants identified

First Author, Year	Mutation (HGVS)	Protein Change	Mutation Type	Affected Domain (LIM)	Isoform(s) Involved
Binder et al., [15]	c.672 C > G	p.(Cys224Trp)	Missense	LIM 4	FHL1A
	c.736 C > T	p.(His246Tyr)	Missense	LIM 4	FHL1A
	c.838G > A	p.(Val280Met)	Missense	Post LIM	FHL1A and B
	c.688 + 1G > A	p.(Ala168Glyfs*195)	Frameshift	LIM 3	FHL1A and B
Borch et al., [20]	NC_000023.11:g.(?_136209656)_ (136211609_? )del (Δexon8)	p.(?)	Deletion→ exon8	LIM 4	All isoform
	c.402_406del;	p.(Gln134Hisfs*9)	Frameshift	LIM 3–4	
	NC_000023.11:g.(?_136206005)_ (136209540_? )del (Δexon3-7)	p.(?)	Large exonic deletion→ exon3-7	LIM 1–4	
D’Arcy et al., [8]	c.764G > C	p.(Cys255Ser)	Missense	LIM 4	FHL1A
		p.(Ala322Pro)		RBP-Jk domain	FHL1B
		p.(Ala193Pro)		RBP-Jk domain	FHL1C
Finch et al., [21]	chrX: 135288965_135291028 [GRCh37]mat	p.(?)	Large exonic deletion→ exon4-6	LIM 1–4	All isoforms
Friedrich et al., [2]	c.134delA	p.(Lys45Serfs)	Frameshift	Pre-LIM	All isoform
	c.459 C > A	p.(Cys153*)	Nonsense	LIM 3 - LIM 4	All isoform
	c.827G > C	p.(Cys276Ser)	Missense	LIM 4	FHL1A
	c.823G > A	p.(Asp275Asn)	Missense	Post LIM	FHL1A
	c.441 C > T	p.(Asp147Asp)	Synonimous	LIM 2	FHL1A
Gaertner-Rommel et al., [22]	c.267 C > A	p.(Cys89*)	Nonsense	LIM 1	All isoforms
Gallego-Delgado et al., [23]	c.468_469insC	p. (Lys157Glnfs*37)	Frameshift	LIM 2–3	FHL1A and B
Giucă et al., [24]	c.157-1G > A	p.(?)	Splice-site variant	Pre-LIM region	All isoforms
Gossios et al., [25]	c.592 C > T	p.(Gln198*)	Nonsense	LIM 3	FHL1A and B
Gueneau et al., [13]	c.625T > C	p.(Cys209Arg)	Missense	LIM 2–3	FHL1A and B
	c.817dup	p.(Cys273Leufs*11)	Frameshift	LIM 4	FHL1A
	c.332_688del	p.(Gly111_Thr229delinsG)	Large in frame deletion → exon 5–6	LIM 3	FHL1A and B
	c.332_501del	p.(Asp112Phefs*51)	Frameshift	LIM 2	FHL1C
	c.469_470delAA	p.(Lys157Valfs*36)	Frameshift	LIM 2	FHL1A and B
		p.(Lys157Valfs*62)	Frameshift	LIM 2	FHL1 C
	c.841T > G	p.(281Glu)	Stop-loss	LIM3	FHL1A
	c.371_372delAA	p.(Lys124Argfs*6)	Frameshift	LIM 2	All isoforms
	c.827G > A	p.(Cys276Tyr)	Missense	LIM 2	FHL1A
Hartmannová et al., [9]	c.599_600insT	p.(Phe200fs32*)	Frameshift	LIM 3	FHL1A and B
Knoblauch et al., [11]	c.625T > C	p.(Cys209Arg)	Missense	LIM 2–3	FHL1A and B
Komagamine et al., [26]	c.310T > C	p. (Cys104Arg)	Missense	LIM 2	All isoforms
López Blázquez et al., [27]	c.144dupT	p.(Asp49*)	Frameshift and Premature Stop Codon	Pre-LIM	All isoform
Nagaraj et al., [28]	Δexon7	p.(?)	Deletion→ exon 7	LIM 4	All isoform
Pen et al., [29]	c.502–2 A > T	p.(?)	Splice-site (acceptor)	LIM 3	Absence of FHL1A and the abundance of FHL1C
Quadrelli et al., [18] (updated Xue [19])	c.502–2 A > G	p.(?)	Splice-site (acceptor)	LIM 3	Absence of FHL1A and the abundance of FHL1C
Schreckenbach et al., [16]	c.449G > C	p.(Cys150Ser)	Missense	LIM 2	FHL1A and B
San Román et al., [33]	c.764G > C	p.(Cys255Ser)	Missense	LIM 4	FHL1A
		p.(Ala322Pro)		RBP-Jk domain	FHL1B
		p.(Ala193Pro)		RBP-Jk domain	FHL1C
Tiffin et al., [30]	c.831 − 20_1017 + 395del602ins84	p.(Ala168Glyfs*175)	Deletion→ exon 6	LIM 3	FHL1A and B
Willis et al., [31]	Not mentioned	p.(?)	Deletion	All domain	All isoforms
Zhang BQ et al., [32]	c.542G > A	p.(Trp181*)	Nonsense	LIM 3	FHL1A and B

*FHL1* Four-and-a-Half LIM Domains 1, *HGVS* Human Genome Variation Society

Nonsense mutations, such as c.267 C > A, p.(Cys89*) and c.592 C > T, p.(Gln198*), were generally associated with more severe phenotypes and earlier disease onset, occasionally culminating in SCD [13, 22]. The c.267 C > A variant, for example, occurred de novo in a patient with HCM and resulted in absent protein expression in both cardiac and skeletal muscle, consistent with nonsense-mediated decay [22]. Frameshift mutations, such as c.469_470delAA, often co-occurred with mixed cardiac and skeletal muscle features [13]. Similarly, large in-frame deletions, notably c.332_688del affecting LIM2–4 encoding exons, were associated with combined phenotypes [13].

Canonical splice site variants (e.g., c.157-1G > A, c.502–2 A > G) further broadened allelic diversity and highlighted the importance of proper mRNA processing in *FHL1*-related diseases [18, 19, 24]. Most pathogenic variants selectively impacted the *FHL1A* isoform, the predominant cardiac isoform, whereas others, such as c.332_688del, affected multiple transcripts [13]. Notably, a large proportion of pathogenic variants clustered within the LIM2 and LIM3 domains (Fig. 2), including recurrent cysteine substitutions such as c.449G > C, p.(Cys150Ser) (classified as pathogenic) and c.625T > C, p.(Cys209Arg) (classified as likely pathogenic), as well as variants like c.371_372delAA, p.(Lys124Argfs*6), c.469_470delAA, p.(Lys157Valfs*36), and c.592 C > T, p.(Gln198*) [11, 13, 15, 16, 25]. These mutations predominantly affected the FHL1A isoform and were consistently associated with cardiomyopathy phenotypes across several cohorts (Table 2). Variants in LIM4, such as c.764G > C, p.(Cys255Ser), and pre-LIM or splice-site mutations, including c.144dupT, p.(Asp49*) and c.502–2 A > G, further broadened the mutational spectrum but were less frequent [8, 18, 27].

### Cardiac phenotype

**Fig. 3:**
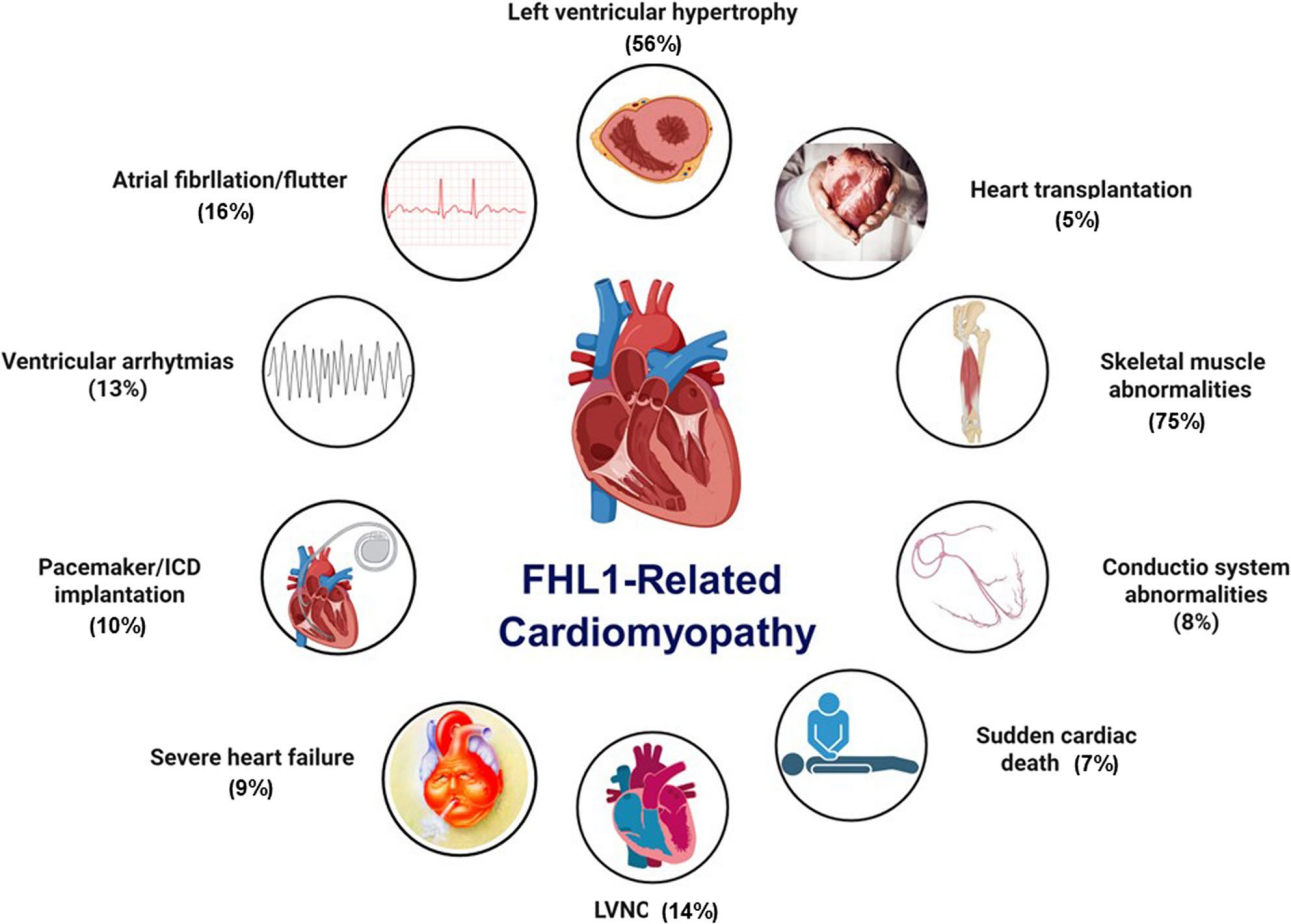
Clinical manifestations and outcomes in patients with FHL1 mutation ICD, implantable cardioverter-defibrillator; LVNC, left ventricular non-compaction

Among the 114 mutation carriers identified, cardiac abnormalities were documented in 108 (95%), while a minority of carriers, mainly female relatives, showed no overt cardiac involvement (Fig. 3). LVH was the most common structural finding, present in 64 individuals (56%), with a significant proportion exhibiting HCM (33 subjects) [2, 8, 9, 11, 13, 15, 16, 18, 20–25, 27–30, 32, 33]. When reported, maximal wall thickness ranged from 12 to 27 mm, with a median of 18 mm [15–19, 23, 27]; in the largest cohort, the mean value was 16.0 ± 1.4 mm [15]. A spongiform myocardial pattern or non-compaction phenotype was observed in 16 patients (14%) Diastolic dysfunction was common, reported in 64% of males in the largest series, and restrictive physiology was described in smaller cohorts, although the severity of diastolic impairment was not consistently quantified across studies [15, 16, 33].

Abnormal electrocardiogram (ECG) was reported in 58 patients (51%). Atrial fibrillation or flutter occurred in 18 (16%), while ventricular arrhythmias, including both sustained and non-sustained ventricular tachycardia (VT), were described in 15 patients (13%) [9, 11, 13, 20, 23, 24, 29, 30]. Conduction system abnormalities, such as atrio-ventricular (AV) block, bundle branch block, and sinus node dysfunction, were present in 11 patients (8%) [13, 15, 16, 26, 31]. Device therapy was common: 1 patient (2%) received pacemakers and 9 (8%) were implanted with an ICD, mainly for secondary prevention [8, 9, 11, 13, 20, 23, 24, 30].

### Neuromuscular involvement

Skeletal muscle abnormalities were documented in 85 of the 114 mutation carriers (75%) and were particularly frequent among male patients [2, 8, 9, 11, 13, 15, 16, 18, 20, 21, 23, 24, 26, 28–33]. Reported features included early contractures, rigid spine, scapulo-peroneal or pelvi-peroneal muscle atrophy, and progressive weakness. In several series, muscle hypertrophy coexisted with wasting, reflecting the heterogeneous neuromuscular phenotype [13, 16, 18, 19].

RBM was specifically reported in association with c.449G > C, p.(Cys150Ser), a missense variant affecting a conserved cysteine residue in the second LIM domain [16]. In other FHL1-related myopathies, muscle biopsy findings included cytoplasmic bodies, rimmed vacuoles, dystrophic/myopathic changes, and FHL1-positive aggregates, but classic reducing bodies were not consistently present [11, 13]. Serum CK levels were typically mildly to moderately elevated, with a median of 870 U/L [250–1200], but values varied widely across cohorts. Electromyography, when reported, demonstrated myopathic or mixed patterns [13, 15, 16, 33], whereas muscle MRI provided structural characterization of muscle involvement, including selective muscle degeneration and fatty replacement. Muscle biopsy findings were heterogeneous and included reducing bodies in selected cases, as well as rimmed vacuoles, cytoplasmic bodies, FHL1-positive aggregates, or nonspecific myopathic/dystrophic changes [13, 16, 33].

Among female carriers, skeletal muscle involvement was less consistent. In the largest cohort [15], most women were asymptomatic or showed only mild contractures or diastolic dysfunction. Other series reported isolated cardiac manifestations without overt myopathy [9, 13, 33], while more advanced neuromuscular or cardiac phenotypes were described only rarely [16].

### Genotype–phenotype correlation

Distinct genotype–phenotype patterns emerged based on mutation class and domain specificity (Table 3). Mutations within LIM3 and LIM4, especially c.672 C > G, p.(Cys224Trp), were tightly linked to the classical XMPMA phenotype with severe cardiac hypertrophy, conduction defects, and ventricular arrhythmias [15]. Truncating variants, including c.468_469insC, p.(Lys157Glnfs*37), c.267 C > A, p.(Cys89*), c.144dupT, p.(Asp49*), and c.831 − 20_1017 + 395del602ins84, p.(Ala168Glyfs*175), have been reported in patients with early-onset or aggressive cardiac phenotypes, including marked LVH, ventricular arrhythmias, aborted SCD/SCD, or advanced heart failure, particularly in young males [22, 23, 27, 30].

Conversely, while some missense mutations such as c.672 C > G, p.(Cys224Trp) were consistently associated with the classical XMPMA phenotype, others like c.625T > C, p.(Cys209Arg) and c.827G > A, p.(Cys276Tyr) showed greater heterogeneity [13]. In males, these variants were typically associated with severe cardiac hypertrophy and neuromuscular involvement, while female carriers demonstrated incomplete penetrance with presentations ranging from asymptomatic status to isolated conduction disease or arrhythmias [2, 13, 16, 33]. Notably, a small subset of variants were associated with isolated HCM in the absence of skeletal muscle disease, including c.134delA, p.(Lys45Serfs; pre-LIM) and c.827G > C, p.(Cys276Ser; LIM4) [2], as well as c.599_600insT, p.(Phe200fs32*; LIM3) [2, 9].

**Table 3:** Genotype–phenotype summary of FHL1 mutations: variant-level clinical features

**First Author**,** Year**	**Mutation (HGVS)**	**Isolated cardiac involvement**	**Presentation < 18y**	Marked LVH*	**Ventricular arrhythmias**	**Conduction abnormalities**	**Diastolic dysfunction ≥Grade II**	**LVEF < 50%**
Binder et al., [15]	c.672 C > G	X	NA	X	✔	✔	✔	X
	c.736 C > T	X	NA	X	NA	NA	✔	X
	c.838G > A	X	NA	X	✔	NA	✔	X
	c.688 + 1G > A	X	X	X	NA	NA	✔	X
Borch et al., [20]	NC_000023.11:g.(?_136209656)_ (136211609_? )del (Δexon8)	X	✔	NA	X	X	NA	X
	c.402_406del;	X	✔	X	✔	✔	NA	X
	NC_000023.11:g.(?_136206005)_ (136209540_? )del (Δexon3-7)	X	X	NA	NA	X	NA	NA
D’Arcy et al., [8]	c.764G > C	X	✔	✔	NA	X	NA	X
Finch et al., [21]	chrX: 135288965_135291028 [GRCh37]mat	X	✔	X	X	X	NA	x
Friedrich et al., [2]	c.134delA	✔	✔	✔	NA	NA	NA	NA
	chrX: 135288965_135291028 [GRCh37]mat	X	✔	✔	NA	NA	NA	NA
	c.134delA	✔	X	✔	NA	X	NA	X
	c.459 C > A	✔	X	✔	NA	NA	NA	NA
	c.827G > C	X	X	X	NA	NA	NA	NA
Gaertner-Rommel et al., [22]	c.823G > A	✔	X	✔	NA	X	NA	NA
Gallego-Delgado et al., [23]	c.441 C > T	X	✔	✔	✔	X	✔	X
Giucă et al., [24]	c.267 C > A	X	X	✔	X	X	✔	X
Gossios et al., [25]	c.468_469insC	X	X	X	X	X	X	X
Gueneau et al., [13]	c.157-1G > A	X	✔	X	X	X	NA	✔
	c.592 C > T	X	✔	✔	X	X	NA	X
	c.625T > C	X	✔	X	X	✔	NA	X
	c.817dup	X	✔	X	X	✔	NA	X
	c.332_688del c.332_501del	X	✔	X	X	X	NA	X
		X	✔	✔	X	X	NA	X
	c.469_470delAA	X	✔	X	X	✔	NA	X
	c.371_372delAA	X	X	X	X	X	NA	✔
	c.841T > G	X	✔	X	X	X	NA	✔
Hartmannová et al., [9]	c.371_372delAA	✔	✔	✔	✔	✔	✔	✔
Knoblauch et al., [11]	c.827G > A	X	✔	NA	X	X	NA	X
Komagamine et al., [26]	c.599_600insT	X	X	X	NA	✔	NA	NA
López Blázquez et al., [27]	c.625T > C	✔	✔	✔	✔	X	NA	X
Nagaraj et al., [28]	c.310T > C	X	✔	NA	NA	NA	NA	NA
Pen et al., [29]	c.144dupT	X	NA	NA	NA	NA	NA	NA
Quadrelli et al., [18] (updated Xue [19])	Δexon7	X	✔	NA	NA	NA	NA	NA
Schreckenbach et al., [16]	c.502–2 A > T	X	✔	X	NA	X	NA	NA
San Román et al., [33]	c.502–2 A > G	X	✔	✔	✔	✔	✔	✔
Tiffin et al., [30]	c.449G > C	X	✔	NA	NA	NA	NA	NA
Willis et al., [31]	Not mentioned	X	✔	NA	NA	✔	NA	NA
Zhang BQ et al., [32]	c.542G > A	X	X	X	X	X	NA	X

*FHL1 *Four-and-a-Half LIM Domains 1, *HGVS* Human Genome Variation Society,* LVH *Left Ventricular Hypertrophy,* LVEF* Left ventricular ejection fraction

*Maximal Wall Thickness ≥ 20 mm

### Clinical outcomes

Despite variable expressivity, clinical outcomes were often severe (Fig. 3). SCD was reported in eight individuals (7%) across six studies [13, 22, 23, 29, 30, 33], with 3 other subjects experiencing aborted SCD [23, 27, 29]. Severe heart failure requiring hospitalization occurred in 11 patients (9%), most commonly due to advanced diastolic dysfunction and restrictive physiology, although progression to systolic impairment was reported in a subset, particularly with truncating variants [9, 11, 16, 18, 23, 24, 33]. Eight patients (7%) died of heart failure or of respiratory complications secondary to severe skeletal muscle involvement [11, 13, 16, 18–20]. Heart transplantation was performed in six patients (5%) [2, 9, 23, 33], with one reported case of post-transplantation death [9].

The severity of outcomes was influenced by both sex and mutation type. Male carriers of truncating or LIM-domain variants were often reported with earlier onset and more severe cardiac manifestations, whereas the degree of skeletal muscle involvement was heterogeneous. In contrast, female carriers generally had later onset and milder disease, though some experienced isolated cardiac manifestations, including atrial arrhythmias and sudden death [16, 22].

## Discussion

This systematic review identified 114 individuals with clinically relevant *FHL1* mutations across 22 studies, predominantly males (69%) with early-onset cardiomyopathy (median onset 18 years) and frequent skeletal muscle involvement. Adverse cardiac outcomes were common, including ventricular arrhythmias (13%), SCD (7%), and heart transplantation (5%). Conduction abnormalities were also prevalent, often requiring pacemaker implantation.

### Pathophysiological mechanisms

The *FHL1* gene encodes the four-and-a-half LIM domain protein 1, which is expressed predominantly in cardiac and skeletal muscle and exists in three main isoforms: FHL1A, FHL1B, and FHL1C [1]. FHL1A, the most abundant isoform in muscle, plays a critical role in sarcomere assembly, force transmission, and biomechanical signaling through interactions with titin, myosin-binding proteins, and MAPK/ERK signaling [5, 34].

Truncating and missense mutations in *FHL1*, especially those involving cysteine residues within LIM domains, disrupt zinc finger structure and destabilize the protein, often leading to complete loss of FHL1A [13, 22, 35]. While some mutations lead to the formation of intracellular aggregates known as RBM, others do not, suggesting two distinct pathological pathways: RBM-associated myopathy and a “non-RBM” disease spectrum dominated by sarcomeric dysfunction and cardiomyopathy [16, 35]. Animal models and in vitro studies confirm that FHL1 deficiency impairs mechanical stress response and titin phosphorylation, contributing to myocardial stiffness and arrhythmogenic remodeling [5, 36].

### Comparison with other genetic cardiomyopathies

Clinically, FHL1-related disease overlaps with other neuromuscular-cardiac syndromes such as those associated with *LMNA*, *DES*, and *PRKAG2*. Like *LMNA* mutations, *FHL1* variants confer a high risk of conduction defects, arrhythmia and SCD, frequently requiring early pacemaker or ICD implantation [37]. However, unlike LMNA, in which cardiac involvement often predominates, FHL1-related disease often includes skeletal myopathy and respiratory muscle involvement, although the extent of these manifestations may be variably reported across studies [13]. *FHL1* mutations result in a unique combination of HCM and neuromuscular signs. Histologically, *DES* mutations may mimic *FHL1-*related disease by producing cytoplasmic aggregates, but their pathogenic mechanism involves intermediate filament disorganization rather than disruption of LIM domain–mediated protein interactions [38]. PRKAG2 mutations, in contrast, cause a glycogen-storage cardiomyopathy that phenocopies hypertrophic cardiomyopathy, typically presenting with left ventricular hypertrophy, pre-excitation, and conduction system disease. However, PRKAG2-related cardiomyopathy lacks the neuromuscular manifestations and respiratory complications characteristic of FHL1 mutations, and histologically it is marked by intracellular glycogen accumulation rather than sarcomeric disarray or reducing bodies [39]. Taken together, these distinctions place FHL1 at a unique intersection of cardiac and neuromuscular disease, underlining the importance of its consideration in patients with unexplained HCM accompanied by contractures, rigid spine, or respiratory involvement.

### Clinical implications and management

Recognition of the FHL1-related cardiomyopathy phenotype is essential for timely diagnosis and tailored management. Although no disease-specific guidelines exist, current recommendations for HCM and inherited arrhythmogenic disorders should be followed, with adaptation for the unique features of FHL1-associated disease [40].

FHL1-related disease often manifests in young males (frequently within the first three decades of life) with unexplained LVH, ECG abnormalities, or conduction disturbances, premature atrial and/or ventricular arrhythmias, and a family history of cardiomyopathies and/or sudden death with non male-to-male transmission [16]. Cardiac MRI findings such as mid-myocardial fibrosis or spongiform appearance are suggestive, especially in patients with preserved ejection fraction but reduced myocardial strain [15]. Systemic red flags include scapuloperoneal weakness, joint contractures, rigid spine, and elevated CK levels, present in 70–90% of males [16]. Muscle biopsy may reveal reducing bodies, fiber size variability, or rimmed vacuoles, further supporting the diagnosis [5, 14, 41].

In female carriers, isolated cardiac manifestations such as arrhythmias or conduction system disease may occur, often in the absence of neuromuscular symptoms, highlighting the need for family screening and sex-specific surveillance strategies [42].

Given the high prevalence of arrhythmias, baseline 12-lead ECG should be obtained in all patients, and extended rhythm surveillance with Holter monitoring or, when appropriate, event/loop recorders are advised in symptomatic individuals or those with a family history of SCD. Exercise testing may unmask chronotropic incompetence or ventricular ectopy. Decisions regarding ICD placement should be individualized but considered in patients with sustained ventricular arrhythmias, syncope, or a family history of SCD. Pacemaker therapy may be required for progressive atrioventricular block or sinus node dysfunction.

**Fig. 4:**
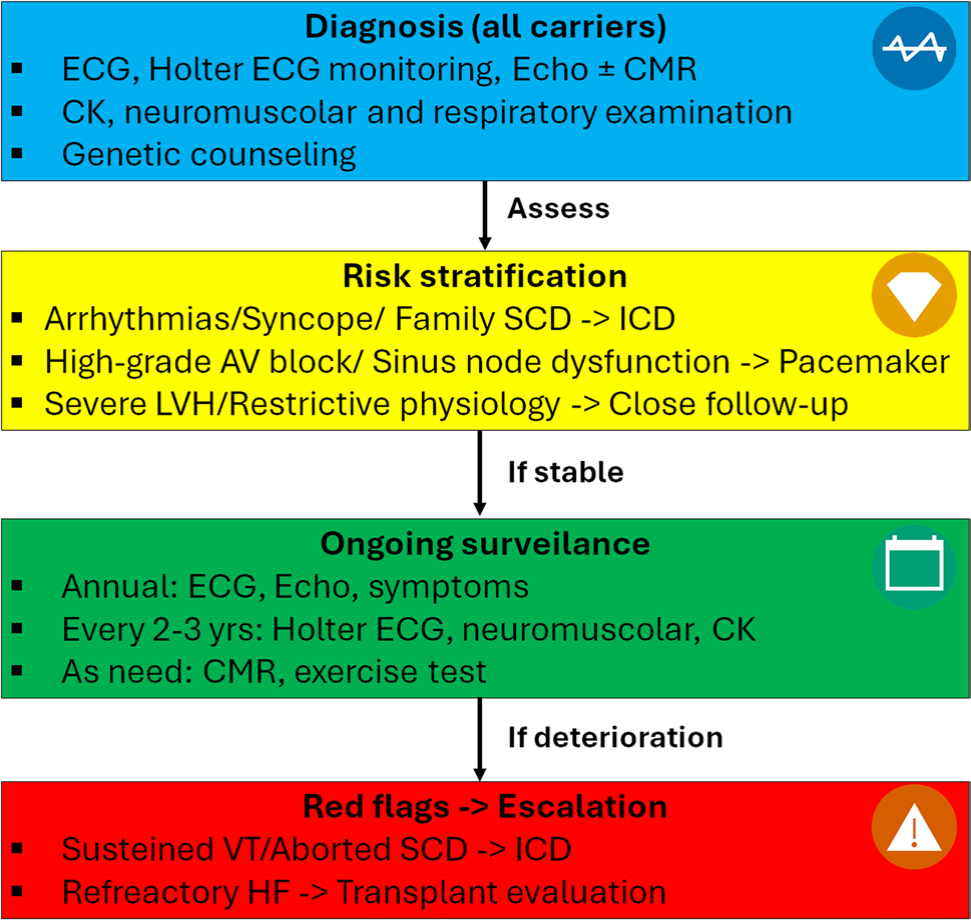
Proposed stepwise diagnosis and follow-up algorithm for FHL1 cardiomyopathies. The pathway integrates baseline rhythm screening, imaging, and neuromuscular/respiratory assessment, followed by risk based device decisions and structured surveillance, with explicit escalation triggers for arrhythmias, respiratory decline, and advanced heart failure. AV block, atrioventricular block; CK, creatine kinase; CMR, cardiac magnetic resonance; ECG, electrocardiogram; HF, heart failure; ICD, implantable cardioverter defibrillator; LVH, left ventricular hypertrophy; SCD, sudden cardiac death; VT, ventricular tachycardia

To ensure early diagnosis and optimize clinical management in patients with *FHL1* cardiomyopathies a multidisciplinary approach is recommended, in particular a close collaboration with the neurologist is critical to assess muscle strength, respiratory function, and bulbar symptoms, as cases of restrictive pulmonary impairment and dysphonia have been documented [13]. While heart failure is often initially mild, some patients progress to advanced disease, and heart transplantation has been reported in several cases. To operationalize these recommendations, we provide a pragmatic, stepwise management algorithm for FHL1 cardiomyopathies that integrates rhythm surveillance, phenotype-directed imaging, multidisciplinary neuromuscular assessment, and risk-based device and HF referrals (Fig. 4).

### Limitations

This review has several limitations. Most included studies were retrospective case reports or small case series, with inherent risks of selection and publication bias. Cardiac phenotyping was often incomplete, particularly in asymptomatic relatives, and skeletal muscle data as well as long-term follow-up were inconsistently reported. Genotype–phenotype correlations could not be established for all variants due to limited number of carriers. In addition, some individuals carried additional pathogenic or likely pathogenic variants in other genes (e.g., in *MYBPC3*) confounding phenotype attribution [22]. Some relevant studies may not have been identified, particularly those in which cardiac involvement was not explicitly reported in the title or abstract. However, the use of predefined and reproducible screening criteria was intended to ensure methodological rigor and minimize subjective selection bias. By restricting inclusion to studies with clinically meaningful and extractable cardiac data, milder or subclinical cardiac involvement may be underrepresented. Moreover, excluding studies without reported cardiac involvement may limit the assessment of the temporal evolution of disease, as cardiac manifestations can emerge at later stages. Consequently, neuromuscular features described in this review should be interpreted within a cardiac-selected population and may not fully reflect the entire phenotypic spectrum of FHL1-related disorders. Although efforts were made to avoid duplication, partial overlap of patient populations across studies cannot be entirely excluded. Despite these limitations, the systematic and detailed extraction of data across multiple studies provides a robust synthesis of the current clinical and genetic landscape of *FHL1*-related cardiomyopathy.

### Future Directions

Prospective registries and collaborative networks are needed to better define the clinical spectrum and natural history of FHL1-related cardiomyopathies. Future studies should aim to correlate specific genotypes and isoform involvement with the severity of cardiac and skeletal manifestations. In vitro and animal model research is essential to dissect the molecular mechanisms underlying FHL1 dysfunction and to explore targeted therapies. Given the phenotypic overlap with other cardiomyopathy–myopathy syndromes, early use of gene panels or whole-exome sequencing should be considered in patients with unexplained cardiomyopathy and neuromuscular features.

## Conclusions

*FHL1* mutations are associated with a heterogeneous spectrum of cardiac phenotypes, including cardiomyopathy, conduction disease, and arrhythmic risk. These findings support the inclusion of *FHL1* in cardiomyopathy gene panels and highlight the importance of a multidisciplinary approach. Early genetic diagnosis, particularly in young patients with unexplained cardiomyopathy or arrhythmias, may improve clinical management and enable appropriate family screening. These results should be interpreted in the context of a cardiac-focused analysis and warrant confirmation in prospective studies including broader *FHL1* populations.

## Data Availability

The data that supports the findings of this study are available on request from the corresponding author.

## Abbreviations

CK: Creatine Kinase
EDMD: Emery–Dreifuss Muscular Dystrophy
FHL1: Four and a Half LIM Domains 1
HCM: Hypertrophic Cardiomyopathy
ICD: Implantable Cardioverter Defibrillator
LVH: Left Ventricular Hypertrophy
MRI: Magnetic Resonance Imaging
PRISMA-P: Preferred Reporting Items for Systematic Reviews and Meta–Analyses Protocols
RBM: Reducing Body Myopathy
SCD: Sudden Cardiac Death
XMPMA: X–linked Myopathy with Postural Muscle Atrophy.
